# Epidermal Expression of Neuropilin 1 Protects Murine keratinocytes from UVB-induced apoptosis

**DOI:** 10.1371/journal.pone.0050944

**Published:** 2012-12-10

**Authors:** Anna Riese, Yvonne Eilert, Yvonne Meyer, Meral Arin, Jens M. Baron, Sabine Eming, Thomas Krieg, Peter Kurschat

**Affiliations:** 1 Department of Dermatology and Venerology, University Hospital of Cologne, Cologne, Germany; 2 Department of Dermatology and Allergology, RWTH Aachen, Aachen, Germany; University Hospital Hamburg-Eppendorf, Germany

## Abstract

**Background:**

Neuropilin 1 (NRP1) is expressed on several cell types including neurons and endothelial cells, where it functions as an important regulator in development and during angiogenesis. As a cell surface receptor, NRP1 is able to bind to members of the VEGF family of growth factors and to secreted class 3 semaphorins. Neuropilin 1 is also highly expressed in keratinocytes, but the function of NRP1 in epidermal physiology and pathology is still unclear.

**Methods and Results:**

To elucidate the role of NRP1 in skin in vivo we generated an epidermis-specific neuropilin 1 knock out mouse model by using the Cre-LoxP-System. Mice were viable and fertile and did not display any obvious skin or hair defects. After challenge with UVB irradiation, we found that deletion of epidermal NRP1 leads to increased rates of apoptosis both in vitro and in vivo. NRP1-deficient primary keratinocytes cultured in vitro showed significantly higher rates of apoptosis 24 hours after UVB. Likewise, there is a significant increase of active caspase 3 positive cells in the epidermis of Keratin 14-Cre-NRP1 (−/−) mice 24 hours after UVB irradiation. By Western Blot analysis we could show that NRP1 influences the cytosolic levels of Bcl-2, a pro-survival member of the Bcl-2 family. After UVB irradiation the amounts of Bcl-2 decrease in both protein extracts from murine epidermis and in NRP1-deficient keratinocytes in vitro, whereas wild type cells retain their Bcl-2 levels. Likewise, levels of phospho-Erk and Rac1 were lower in NRP1-knock out keratinocytes, whereas levels of pro-apoptotic p53 were higher.

**Conclusion:**

NRP1 expression in keratinocytes is dispensable for normal skin development. Upon UVB challenge, NRP1 contributes to the prevention of keratinocyte apoptosis. This pro-survival function of NRP1 is accompanied by the maintenance of high levels of the antiapoptotic regulator Bcl-2 and by lower levels of pro-apoptotic p53.

## Introduction

Like no other organ the human skin as the outer protective barrier of the body is exposed to a variety of external insults. The hazards can be of either physical, chemical or biological nature. Especially the epidermis, consisting mainly of keratinocytes forming a stratified epithelium, is continuously exposed to environmental hazards. This challenge by carcinogens is reflected by the fact that epithelial skin cancer is the most common neoplastic disease in humans [1]. The main causative agent for non-melanoma skin cancer is environmental UV-irradiation, being accountable for approximately 90% of non-melanoma skin cancer [2].

To prevent skin cancer formation evolution has developed two protective measures. If cell damage is not too severe the DNA repair machinery tries to remove and to replace mutated sequences. In the case of more extensive damage apoptosis is activated. It should be kept in mind that during terminal differentiation keratinocytes undergo a specialized form of programmed cell death called cornification [3]. Although this process is different from apoptosis, it shares several similarities and utilizes many common intracellular mechanisms. Therefore, the main components for programmed cell death are permanently present in epidermal keratinocytes and need to be suppressed during early phases of differentiation in the basal and suprabasal layers. This prevention of premature cell death depends on the permanent stimulation with mitogens like EGF, KGF or HGF. Accordingly, the highest expression of the required receptor tyrosine kinases is found in basal layers of the epidermis. In summary, epidermal keratinocytes depend on efficient growth factor stimulation to ensure a balance between proliferation and cell death for the maintenance of skin homeostasis.

Neuropilins (NRP1 and NRP2) are type I transmembrane receptors with a size of approximately 130 kDa. They were discovered in the nervous systems as receptors for secreted class 3 semaphorins [4]. Upon ligand binding their main function is to mediate repulsive signals for outgrowing axons and migrating neurons. The induced signal transduction events are dependent on the interaction of NRPs with plexins, which were shown to constitute the signal transducing subunit of the receptor complex. Later on it was found that neuropilins are also expressed on endothelial cells [5]. On these cells they act as isoform-specific coreceptors for members of the VEGF-family of growth factors, including VEGF-A_165_ or PlGF-2. Following ligand binding the signal is mediated via VEGF-receptor tyrosine kinases, mainly VEGF-R2, which results in increased angiogenesis [6].

The relevance of NRPs for development is underlined by the observation, that the complete knock-out for NRP1 in mice is embryonic lethal around day E11.5. The phenotype displayed severe vascular and nervous defects [7]. The double knock-out of both NRP1 and NRP2 is characterized by an even more severe phenotype which is embryonic lethal around day E8. This is comparable to the complete knock-out of VEGF [8].

In the immune system, neuropilins were identified as part of the immunological synapse between antigen presenting cells and T cells, enhancing T cell activation [9], [10]. Furthermore, neuropilin receptors were described on different types of cancer cells, and for different tumour entities their expression could be correlated to advanced tumour stages and increased aggressiveness of disease in vivo [11], [12]. For MDA-MB 231 mammary carcinoma cells it was shown that VEGF-A_165_ acts as an autocrine survival factor which inhibits apoptosis in a NRP-dependent manner [13].

The expression of NRP1 by human epidermal keratinocytes in vitro and in epidermal layers in vivo was already demonstrated in 2000 by Gagnon and coworkers and later confirmed by another publication [14], [15]. We could show that this expression is positively regulated by growth factors like EGF or HB-EGF and that NRP1 on keratinocytes serves as a receptor for Sema3A, mediating the inhibition of keratinocyte migration [16]. Nevertheless, the precise function of neuropilin expression in epidermal keratinocyte physiology remains largely unclear.

To further elucidate the role of neuropilins in epidermal keratinocytes and since the complete knock-out of NRP1 shows early embryonic lethality, we generated epidermis-specific NRP1 knock-out mice, using the Cre-loxP-technology. A function for neuropilins in the regulation of apoptosis has already been demonstrated for neurons under hypoxic conditions and for cancer cells. UVB irradiation is one of the most potent inductor of keratinocyte apoptosis in vitro and in vivo. Therefore, we studied the effects of NRP1-deficiency on programmed cell death after UVB irradiation and analyzed the intracellular signal transduction events.

## Materials and Methods

### Ethics statement

All necessary permits were obtained for the described studies. The use of human skin tissue from the tissue collection of the Dept. of Dermatology, University Hospital of Cologne for immunohistochemical stainings was approved by the Ethics committee of the University of Cologne, Cologne, Germany. The use of mice as animal models was approved by the local authorities of the state of Northrhine Westfalia (Landesamt für Natur, Umwelt und Verbraucherschutz NRW, permission number 50.203.2-K 23, 18/06). The permission included the generation of conditional knock out animals and the protocols for UVB irradiation. All experimental procedures causing discomfort of the animals were carried out under general anaesthesia.

### Generation of keratinocyte-specific conditional NRP1 knock out mice and isolation of primary keratinocytes

Mice (C57/Bl6 background) with floxed exon 2 of the *nrp* gene were obtained from Jackson Lab [17]. They were crossed to a mouse line which expresses Cre recombinase under the control of the Keratin 14 promoter [18]. Genotyping were performed by PCR with genomic DNA isolated from tails. Primer sequences were: Cre-for GAC GGA AAT CCA TCG CTC GAC CAG and Cre-rev GAC ATG TTC AGG GAT CGC CAG GCG, NRP1-for AGG CCA ATC AAA GTC CTG AAA GAC AGT CCC and NRP1-rev AAA CCC CCT CAA TTG ATG TTA ACA CAG CCC.

Primary keratinocytes were isolated from the skin of newborn mice and cultured on collagen type-1 (CellSystems) coated dishes in F12, adenine, DMEM (FAD) medium containing 50 mM Ca^2+^. Keratinocytes were used until passage 4.

### UVB-irradiation of keratinocytes or animals and cell cycle analysis

Keratinocytes in vitro were irradiated with 20 mJ/cm^2^ in a BioSun machine (2×20watt) (Vilber Lourmat) with an emission peak of 312 nm. UVB irradiation of mice was done using a Waldmann TV-4 device. Back hair of mice in telogen hair cycle phase was shaved. Then the anterior region of the back was exposed to a single dose of 1000 mJ/cm^2^ of UVB under general anaesthesia. The unirradiated posterior region was used as a control. Mice were killed at different time points after UVB irradiation and skin samples (1 cm×0.5 cm) were taken from paramedian part of posterior and anterior back skin regions.

To analyze the phase of the cell cycle of cultured keratinocytes by measurement of DNA content, cells were harvested by trypsinization, fixed with cold 70% ethanol, washed with PBS and stained with propidium iodide (PI). Flow cytometric analysis was performed using a FACSCalibur (BD Biosciences). All experiments were performed at least three times independently.

### Western blotting and Quantitative real-time RT-PCR

Cells were solubilized in RIPA buffer (1% Nonidet P-40, 0.5% sodium deoxycholate, 0.1% SDS in PBS) containing 1 mM PMSF, 10 µg/ml aprotinin, and 10 µg/ml leupeptin at 4°C for 20 min. After centrifugation at 10,000× *g* for 15 min, the protein concentrations of supernatants were determined by using the BCA Protein Assay Reagent (Pierce Chemical Company). Samples containing 30 µg of proteins were boiled for 5 min in Laemmli buffer. Primary antibodies were directed against phospho-ERK (Thr202/Tyr204, Cell Signaling), phospho-p38 (Thr180/Tyr182, Cell Signaling), phospho-Akt (Ser473, Cell Signaling), Bcl-2 (Cell Signaling), phospho-p53 (Ser20, Cell Signaling), total ERK, total p38, total Akt, Mcl-1 (all Cell Signaling) or beta-actin (SantaCruz).

Quantitative Real-Time PCR was performed with a 7300 Real Time PCR system (Applied Biosystems) using PowerSYBR Green following the manufacturer's protocol. The relative fold-changes in mRNA expression were calculated according to a ΔΔCt method normalized against the S26 gene. Normalization against glyceraldehyde-3-phosphate dehydrogenase (GAPDH) was used for confirmation and led to the same results. The primer sequences were S26-for 5′-GTG CCA TCC ATA GCA AGG TT-3′ and S26-rev 5′-GTG GAG GTC GAG GTG CAG-3′; for Neuropilin 1 5′-TAC CTC ACA TCT CCC GGT TAC C-3′ and 5′-GAA GAT TTC ATA GCG GAT GG-3′. All Western blots and RT-PCRs were repeated at least three times independently with cell or tissue extracts from different animals.

### Histology and immunohistochemical stainings

Immunohistochemistry was performed on paraffin or cryostat sections using monoclonal antibodies against Neuropilin 1, Caspase-3 (Cell Signaling Technology), CD31 (BD Biosciences) and polyclonal antibodies against Keratin 14, Keratin 10 and Loricrin (Covance). Secondary antibodies were labelled with Alexa 488 (Invitrogen). Nuclei were counterstained using propidium iodide. Fluorescent stainings were photographed using a Nikon Eclipse 800 microscope equipped with a DXM1200 digital camera. TUNEL staining was performed on formalin-fixed and paraffin-embedded sections by using a staining kit (Promega, G3250) following the manufacturer's instructions. For routine histology paraffin-embedded sections were stained with haematoxylin/eosin, according to standard protocols. For each time point and experiment at least three different animals were investigated.

### G-LISA and statistical analysis

For the determination of Rac1 activation a G-LISA (Cytoskeleton) was performed following the manufacturer's instructions. Briefly, cell lysates were made 20 min after UVB irradiation. After adjusting all samples to a protein concentration of 2 mg/ml the samples were analysed in triplicates. The luminometric readout of results was performed using a Victor.

Statistical analysis was performed using GraphPad Prism5 (GraphPad Software). Significance of difference was assessed using unpaired Student's t-test. All data are presented as means ± standard deviation A p-value of less than 0.05 was considered significant. Densitometric analysis of Western blots was performed with ImageJ-software. Protein amounts were normalized against beta-actin. The value for the untreated first lane (no UV light) was set to 1.0. To test for statistically significant differences 3 or more different Western blots were analyzed, a p-value<0.05 was considered significant.

## Results

### Generation and characterization of keratinocyte-specific NRP1 knock-out mice

**Figure 1:**
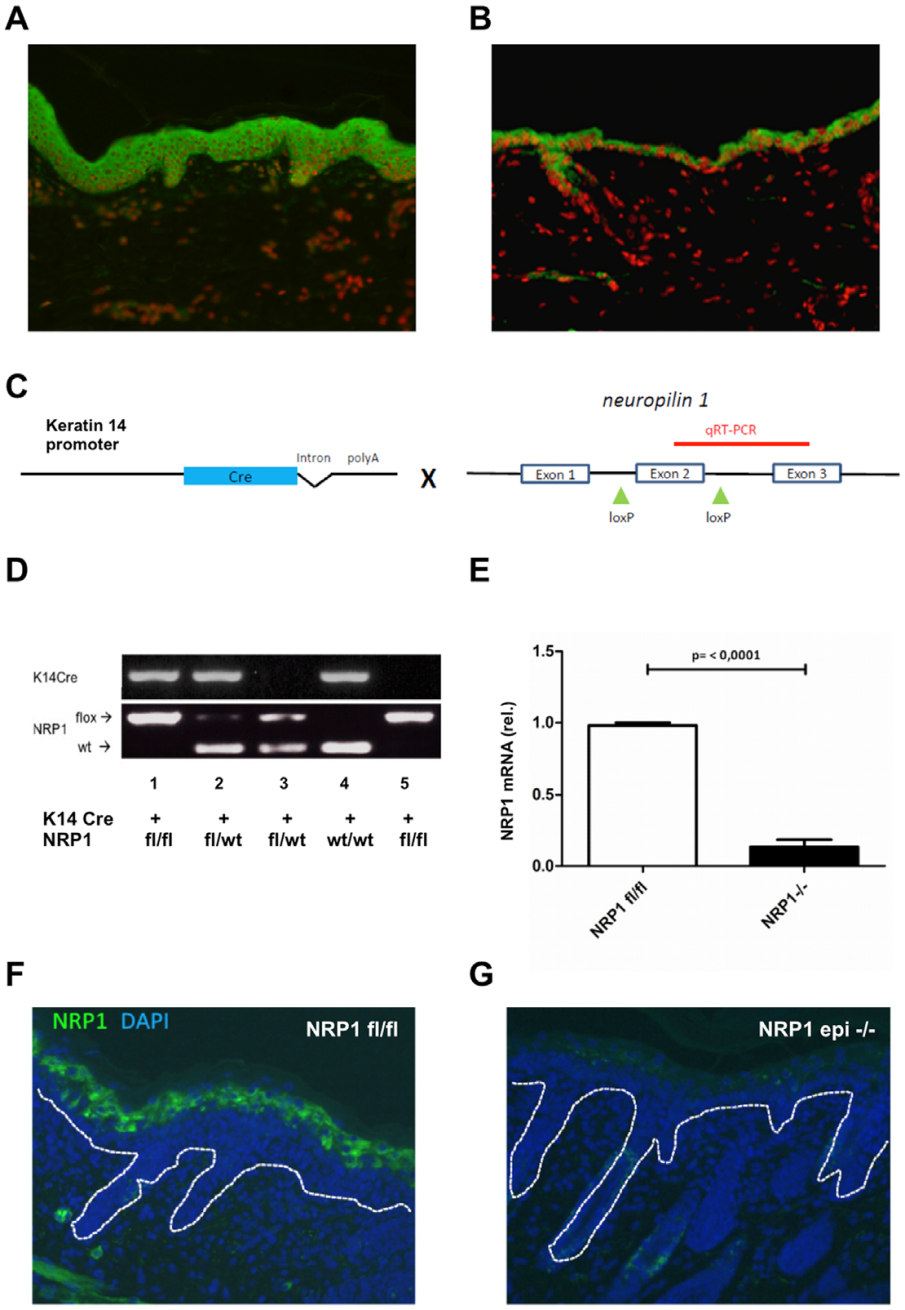
Expression and epidermis-specific deletion of neuropilin 1 in mice. Immunofluorescent staining for NRP1 in cryosections of normal human skin (A) or skin from 8 weeks old mice (B) shows protein expression in all layers of the epidermis, with a tendency towards stronger staining in the suprabasal layers (green fluorescence, nuclear stain in red with propidium iodide). For conditional keratinocyte-specific ablation of NRP1 expression, Keratin 14-Cre mice were crossed with mice homozygous for a mutated NRP1 gene containing loxP sites flanking the exon 2 (C). Genotyping of animals was performed by PCR (D). Keratinocytes isolated from newborn mice were checked by quantitative real time PCR for NRP1 mRNA levels (E). Additionally, NRP1 expression in the epidermis was assessed by immunofluorescent staining and demonstrated absence of detectable NRP1 protein in the epidermis of NRP1epi−/− mice (Figure 1 F, G, green fluorescence, the broken line reflects the epidermal basement membrane).

The strong expression of neuropilin 1 by epidermal keratinocytes has been described before [15], [16]. By immunohistochemical staining we could verify the expression of NRP1 in all layers of the epidermis in human and mouse skin sections (Figure 1 A, B, green fluorescence, nuclear stain in red with propidium iodide). Until now, the function of epidermal NRP1 in physiological and pathological situation is still unclear. Since the total knockout of NRP1 in mice is embryonic lethal around embryonic day 12 [7], we used the Cre-LoxP system to achieve tissue-specific ablation of NRP1 expression. To generate epidermis-specific NRP1 knock out mice, we crossed mice which expressed Cre recombinase under the control of the keratin 14 promoter [18] with mice homozygous for a mutated NRP1 gene containing two loxP-sites flanking the exon 2 (Figure 1 C) [17]. Keratin 14 (K14) is expressed in all keratinocytes of the basal layer of the epidermis. Genotyping was performed by polymerase chain reaction (Figure 1 D). To check for efficient ablation of NRP1 protein expression keratinocytes from newborn mice were isolated and cultured in vitro. As shown by quantitative real time RT-PCR, the detectable amounts of NRP1 mRNA were below 20%, as compared to keratinocytes homozygous for the floxed NRP1 gene but lacking Cre recombinase activity (Figure 1 E). The remaining signal for NRP1 mRNA might be attributable to contamination with fibroblast feeder cells, since primary murine keratinocytes require these cells for cultivation in vitro. Using crysections of newborn mice (day 2 postnatally) we could confirm efficient ablation of NRP1 protein expression in the epidermal layers of conditional knock out mice (Figure 1 F, G). It should be noted that in newborn mice NRP1 expression is most prominent in the upper epidermal layers.

**Figure 2:**
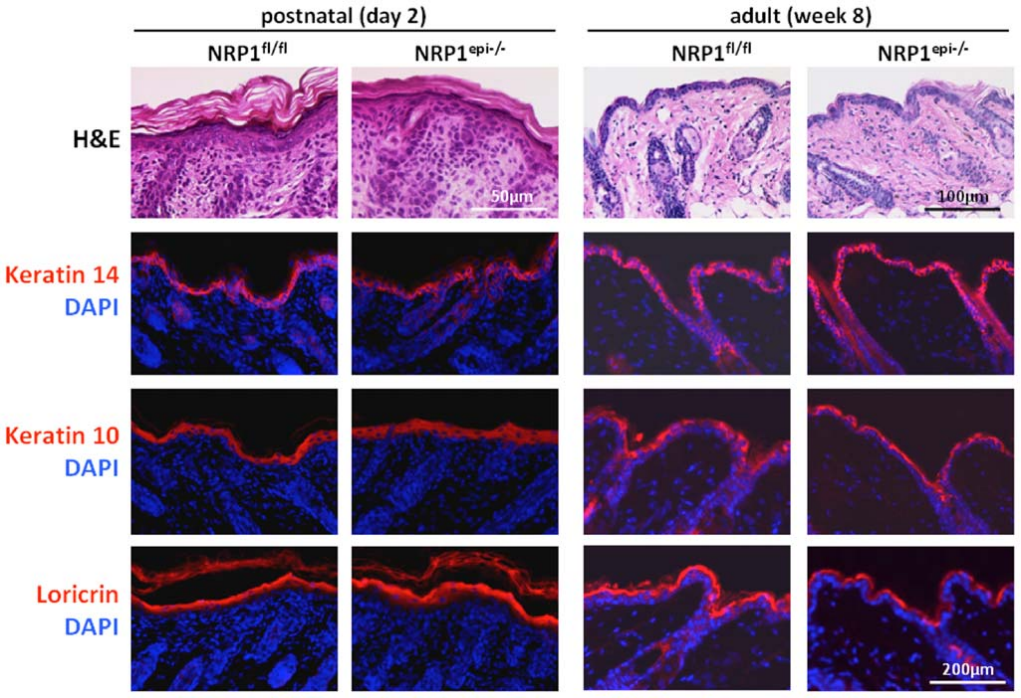
Microscopic characterization of keratinocyte-specific NRP1-deficient mice. Hematoxylin/eosin staining shows no differences in skin architecture between control and NRP1-deficient mice postnatally or in adult mice. Likewise, no changes in the expression of various differentiation markers could be detected. These markers included keratin 14 for basal keratinocytes, keratin 10 for suprabasal keratinocytes and loricrin for late stage differentiation, preferentially in the granular layer.

Mice lacking NRP1 expression in epidermal keratinocytes were viable and fertile and did not display any macroscopically obvious phenotype. To search for more subtle changes, newborn (postnatal day 2) and adult mice (week 8) were investigated by histology and immunofluorescent stainings (Figure 2). Hematoxylin/eosin staining showed a regularly stratified epithelium with ordinary developed hair follicles. To detect defects in keratinocyte differentiation sections were stained with antibodies against the basal cell marker keratin 14, the marker keratin 10 for suprabasal keratinocytes and the late differentiation marker loricrin. No differences between keratinocyte-specific NRP1 knock out animals and control animals could be detected, indicating that NRP1 expression is dispensable for regular epidermal development and differentiation.

### NRP1-deficient keratinocytes show increased apoptosis in vitro and in vivo following UVB irradiation

**Figure 3:**
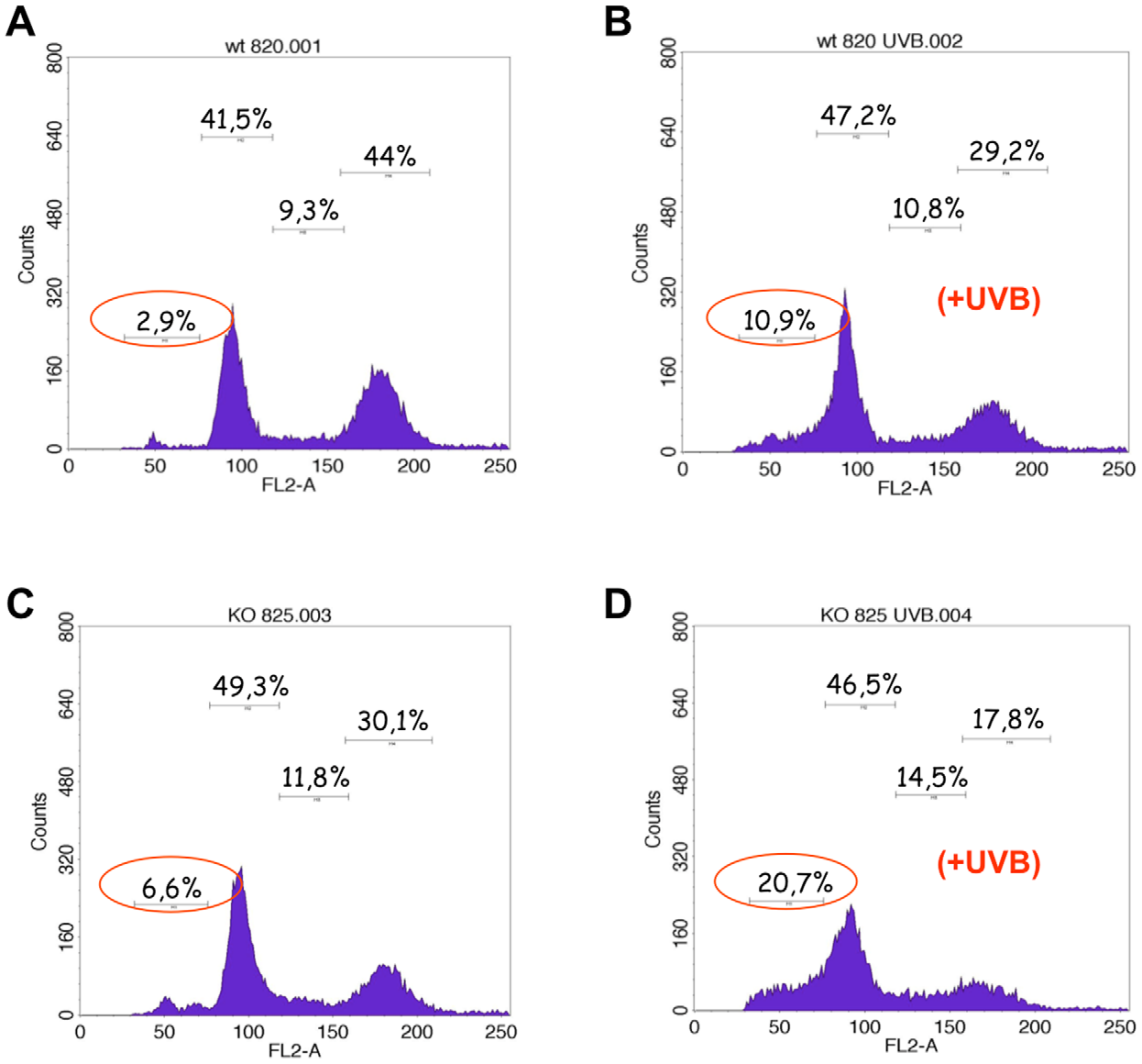
Induction of apoptosis by UVB irradiation in NRP1-deficient keratinocytes and epidermis. Primary murine keratinocytes were isolated from epidermis-specific NRP1-deficient or control animals irradiated with UVB light (20 mJ/cm^2^) in vitro. 24 h after irradiation keratinocytes were harvested and stained with popidium iodide for cell cycle analysis by FACS (A–D). Cells in sub G1 phase were considered to be apoptotic. After UVB treatment 20.7% of NRP1-deficient keratinocytes were apoptotic, whereas only 10.9% of control cells were in subG1 phase. The figure shows one representative example out of three independent experiments.

**Figure 4:**
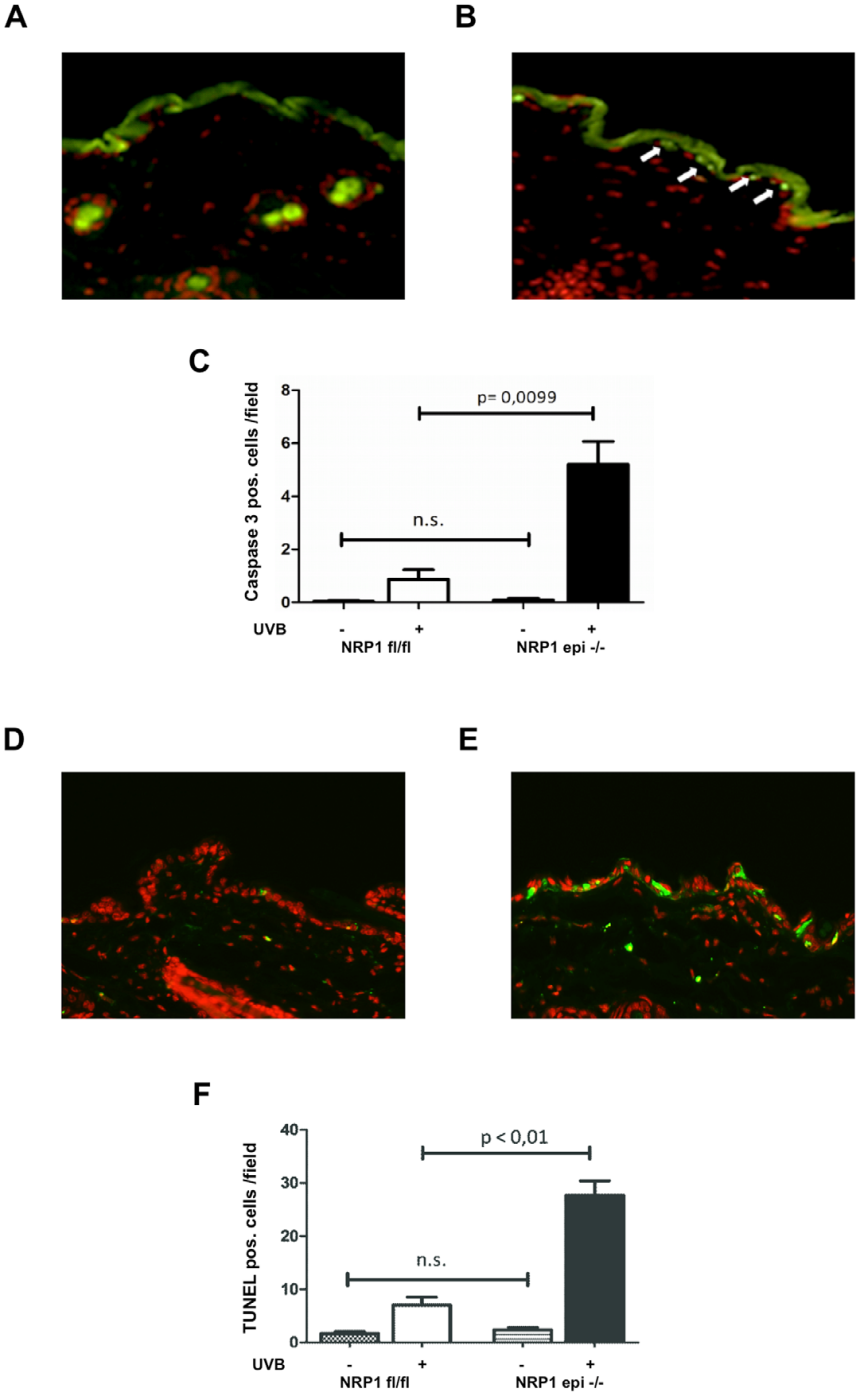
UVB irradiation leads to increased apoptosis in NRP1-deficient epidermis in vivo. To investigate UVB-induced apoptosis in vivo epidermis-specific NRP1 knock out mice and control animals were irradiated with 1 J/cm^2^. 24 hours later skin biopsies were stained for active caspase 3 in control (A) or knock out (B) animals. Apoptotic cells were counted and analyzed for statistically significant differences (C). Additionally, apoptotic cells were stained by TUNEL assays (D, E) and cells were quantified (F). Figure 4 (A to F) shows one representative example out of three independent experiments.

As a cell surface receptor for VEGF-A neuropilin 1 has been demonstrated to prevent apoptosis in different cell types like neurons, cancer cells or embryonic stem cells [13], [19], [20]. The most important inductor of keratinocyte apoptosis in humans is ultraviolet radiation. Since epidermis-specific ablation of NRP1 expression in vivo did not result in a spontaneous phenotype we challenged primary murine keratinocytes in vitro with UVB irradiation. 24 hours after exposure to 20 mJ/cm^2^ of UVB light (peak emission of 312 nm) cells were subjected to cell cycle analysis (Figure 3 A–D). Following propidium iodide staining DNA content was measured by FACS. Prior to UVB exposure the amount of apoptotic cells (subG1 phase) was 2.9% in controls and 6.6% in NRP1-deficient keratinocytes. After UVB irradiation the proportion of apoptotic cells increased to 10.9% in floxed control cells, whereas in NRP1-lacking keratinocytes the amount increased to 20.7%. This experiment was repeated three times independently with primary keratinocytes from different animals. In all three experiments the percentage of apoptotic cells after UVB irradiation was twice as high in NRP1-knock out cells (mean factor 1.9-fold, standard deviation 0.36), as compared to the floxed control cells, although the percentage of apoptotic cells varied between 10% and 25%. To prove that cells in subG1 phase are really apoptotic cells the same experiment was performed with annexinV-staining and FACS analysis, confirming the results demonstrated here. Using the same experimental conditions as described above (24 hours after exposure to 20 mJ/cm^2^ of UVB light) 15.3% of the NRP-1 knock out cells, but only 8.1% of the control cells stained positive for annexinV (data not shown). To further investigate whether this increased susceptibility of NRP1-deficient keratinocytes to UVB-induced apoptosis was also present in vivo epidermis-specific NRP1-knockout mice were irradiated with a single dose of 1000 mJ/cm^2^ of UVB. 24 hours later skin samples were taken and stained for apoptotic cells with an anti-active Caspase 3 antibody (Figure 4 A, B). Following UVB irradiation, higher numbers of positively stained cells were detected in NRP1-deficient epidermis. Quantification revealed an increase of more than 5-fold, as compared to control animals (Figure 4 C). The difference was of high statistical significance (p-value of less than 0.001). Prior to UVB exposure, only few apoptotic cells were detectable, showing no difference in relation to NRP1 expression. To reproduce this important finding in vivo by a different technique, the same experiment was performed with subsequent TUNEL staining (Figure 4 D, E). Again, the result was of high statistical significance (Figure 4 F).

### Analysis of NRP1-dependent signal transduction events in keratinocytes after UVB-treatment

**Figure 5:**
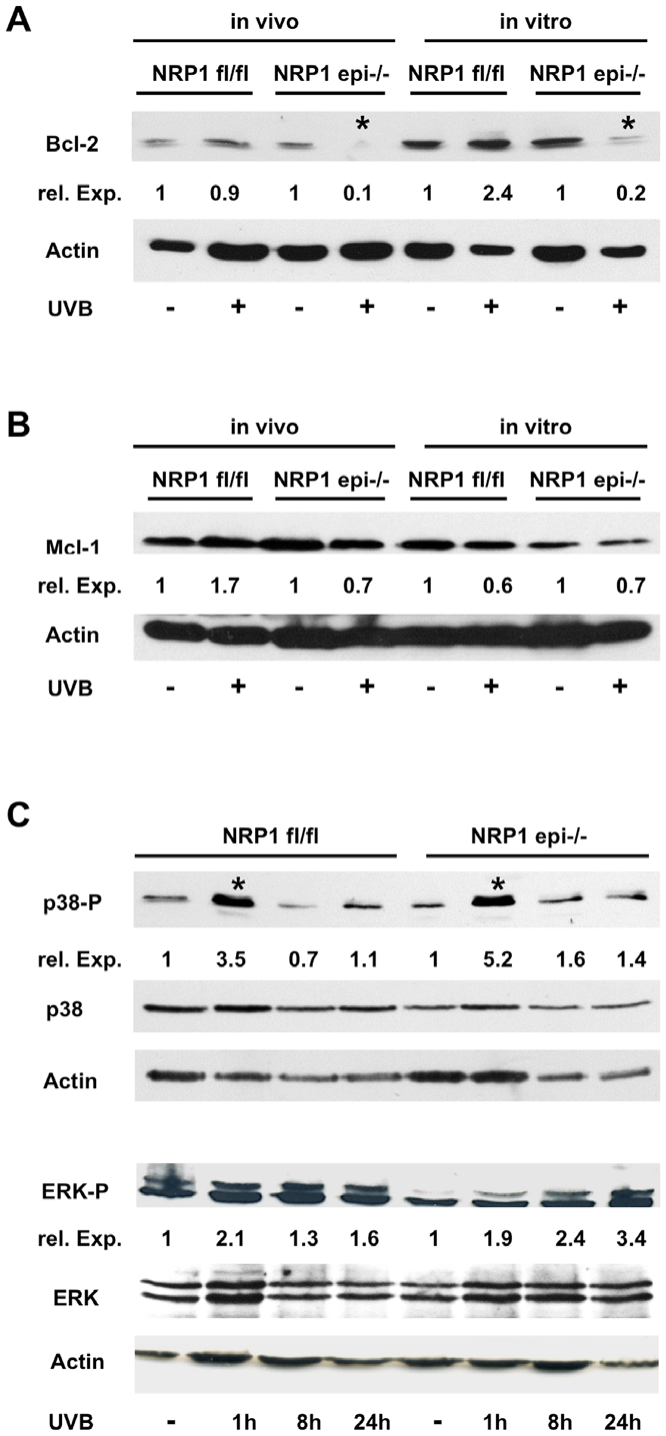
Differential regulation of Bcl-2 in NRP1-deficient and control keratinocytes following UVB irradiation. 24 h after UVB irradition protein extracts from mouse epidermis and from primary keratinocytes cultured in vitro were prepared and analyzed by Western blotting. Prior to UVB treatment the amounts of Bcl-2 are comparable between control and NRP1-deficient samples (A). After UVB application Bcl-2 levels decrease significantly (p<0.05, indicated by an asterisk) in NRP1-deficient epidermis and cells, whereas control tissue and cell extracts show no significant changes in Bcl-2 amounts. No significant changes were observed in the levels of Mcl-1, phospho-p38 (only the induction 1 hour after UVB was significant, but present in control and NRP1-deficient cells) and phospho-Erk (B, C). All Western blots were repeated independently with extracts from tissue or cells from at least three different animals. The relative expression expresses the fold-change in comparison to the non-irradiated control lane.

The anti-apoptotic protein Bcl-2, localized mainly in the outer membrane of mitochondria, has been identified as one of the key regulators of UVB-induced apoptosis in keratinocytes [21]. Overexpression of Bcl-2 in vitro and in vivo leads to decreased programmed cell death of keratinocytes [22]. Therefore, we determined the levels of Bcl-2 protein in epidermis-specific NRP1 knock out mice and control mice following UVB irradiation (Figure 5 A). Prior to UVB exposure epidermal protein extracts from both mice contained comparable amounts of Bcl-2. 24 hours after a single dose of UVB (1 J/cm^2^) Bcl-2 was markedly reduced in epidermal extracts from NRP1-deficient animals, whereas in control mice only a weak reduction was observed. Likewise, in keratinocytes isolated from these animals and cultured in vitro the detectable amount of Bcl-2 decreased stronger in NRP1-deficient cells after irradiation with UVB (24 hours after 20 mJ/cm^2^ UVB). To check for statistical significance, expression levels of Bcl-2 were normalized against beta-actin and 3 different blots were analyzed. Following UVB irradiation the Bcl-2 amount in NRP1-deficient keratinocytes decreased to below 20% of the prior level, which was a significant change. Similar to Bcl-2, Mcl-1 has been described as a major regulator of UVB-induced apoptosis in keratinocytes [23], [24]. Therefore, we analyzed Mcl-1 expression by Western blotting (Figure 5 B). Although there was a tendency towards reduced levels after UVB exposure, this change failed to reach statistical significance.

**Figure 6:**
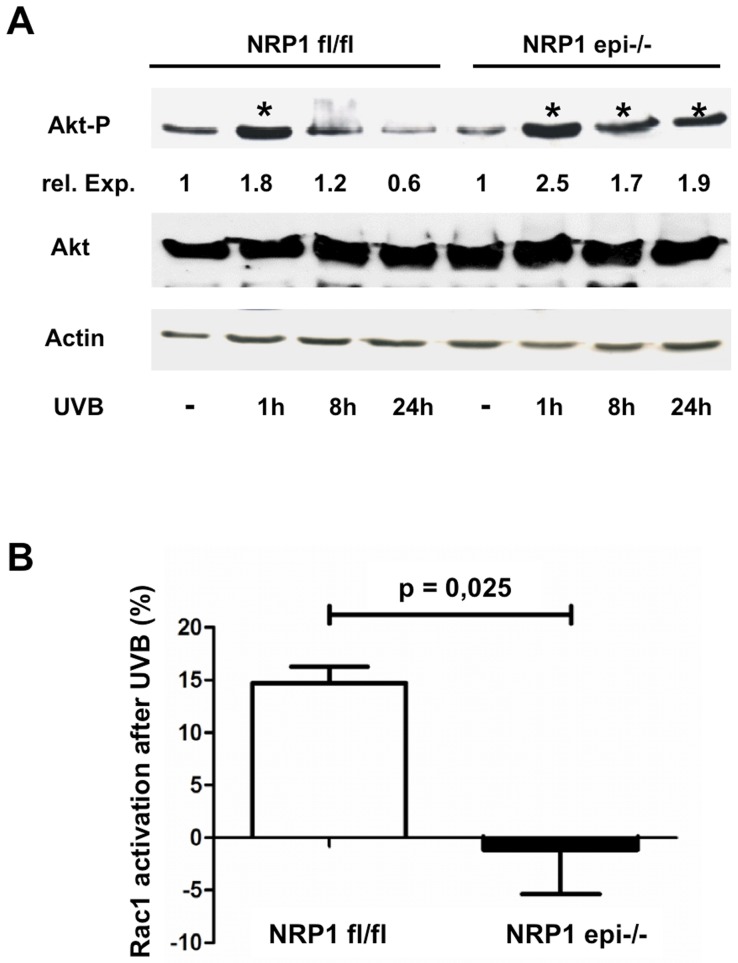
NRP1-deficient keratinocytes are characterized by a lack of Rac1 activation after irradiation with UVB light. Like in Figure 5, keratinocytes or animals were subjected to UVB treatment and protein extracts from mouse epidermis or from primary keratinocytes cultured in vitro were prepared after 24 h and analyzed by Western blotting. Compared to control keratinocytes, phospho-Akt activation is longer persistent in NRP1-deficient cells (A). Additionally, NRP1-knock out keratinocytes did not activate Rac1 after UVB irradiation (B). All Western blots were repeated independently with extracts from tissue or cells from at least three different animals. The relative expression expresses the fold-change in comparison to the non-irradiated control lane.

Next we tried to determine which signal transduction cascades upstream of Bcl-2 expression are altered in NRP1-deficient keratinocytes. The two anti-apoptotic pathways most intensively studied in keratinocytes are the PI3-kinase and MAP-kinase signal transduction cascades [25], [26]. Both can be activated by a variety of growth factors like EGF, VEGF or PDGF, which in turn initiate the phosphorylation of receptor tyrosine kinases. Further downstream both pathways increase Bcl-2 levels [27], [28], [29]. To check for activation of these pathways we measured the phosphorylated forms of Akt, p38 and Erk (Figure 5 C, Figure 6 A). Whereas no changes could be determined for p38 or Erk, activation of Akt was more persistent in NRP1-deficient keratinocytes after UVB exposure. Another factor important for keratinocyte survival is Rac-1 activation [30]. Therefore, we determined Rac1-activation in keratinocytes after UVB irradiation in vitro. As measured by GLISA-technique we could show that control cells activate Rac1 in response to UVB light, whereas NRP1-deficient cells are characterized by the lack of Rac1-activation. This difference was statistically significant. (Figure 6 B).

**Figure 7:**
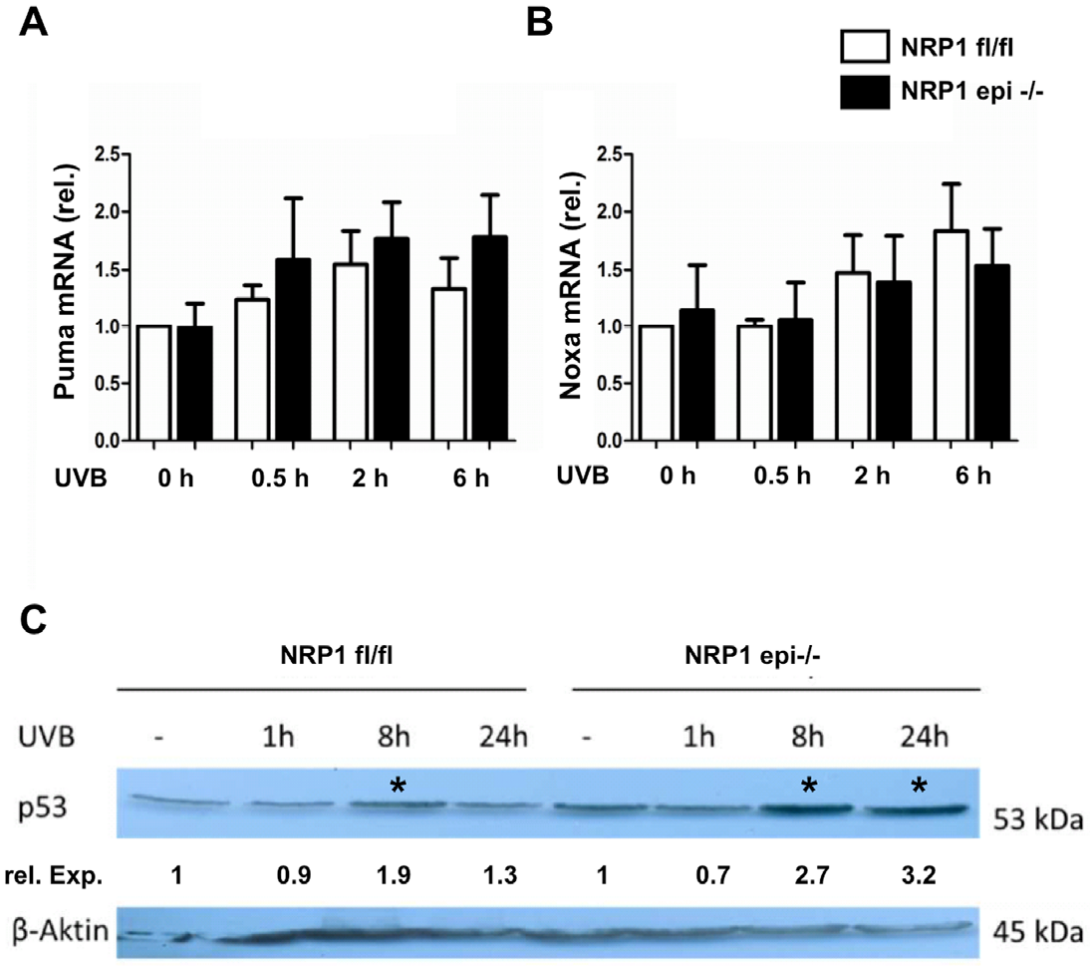
After UVB treatment NRP1-deficient keratinocytes show a stronger activation of p53, as compared to control cells. Following UVB irradiation of keratinocytes in vitro, the expression levels of the pro-apoptotic factors puma and noxa showed a comparable increase in control and NRP1-deficient cells (A, B). In contrast to this, NRP1-knock out keratinocytes are characterized by a stronger and more persistent activation of p53, as reflected by increased phosphorylation of serin residue 20 (C). Quantitative RT-PCR and Western blotting was repeated independently with cells isolated from at least three different animals. The relative expression indicates the fold-change in comparison to the non-irradiated control lane. Significant changes (analysis of 3 blots, p<0.05) are indicated by an asterisk.

The signal transduction pathways described before antagonize apoptosis by increasing or stabilizing Bcl-2. On the other hand, it has been shown that following UVB irradiation Bcl-2 levels can be decreased by increased expression of the pro-apoptotic factors puma and noxa [31]. Therefore, we determined expression in NRP1-deficient and control cells. Following UVB exposure both puma and noxa increase, but there was no difference between keratinocytes expressing or lacking NRP1, at least not within the first 6 hours after UVB irradiation (Figure 7 A, B). Puma and noxa are not regulated by protein phosphorylation, but rather by transcription. They belong to the group of genes upregulated by p53, which in turn is activated by UVB-induced DNA damage in keratinocytes [32]. Therefore, the activation of p53 (phosphorylation of Ser20) was investigated after UVB irradiation. Whereas in control cells only a transient elevation of phospho-p53 was observed with a maximal response 8 hours after UVB, NRP1-deficient keratinocytes showed a stronger and more persistent activation of p53 (Figure 7 C). This difference was statistically significant and corresponds well to the known function of activated p53 as an inducer of keratinocyte apoptosis.

## Discussion

Our study was intended to elucidate the role of NRP1 in epidermal keratinocytes. To investigate the impact of NRP1 in the epidermis under physiological conditions we ablated the expression of NRP1 in keratinocytes. Besides NRP1, a second neuropilin (NRP2) with a similar domain structure has been described. According to previous publications and own unpublished data keratinocytes express not only NRP1, but also NRP2 [14]. On the functional level, the two NRPs have been shown to have both overlapping and specific functions, depending on the cellular context. Additionally, they have distinct binding specificities to the various ligands. For example, NRP1 binds strongly to Sema3A and very weekly to Sema3F, whereas NRP2 shows the opposite behaviour. The question whether or not NRPs have redundant and potentially compensating functions in skin physiology and pathology can not be answered at the moment. Compensation could be one explanation for the lacking spontaneous phenotype in keratinocyte-specific knock out mice. On the other hand, our investigations showed that under stress conditions like UVB irradiation NRP1-deficient and control keratinocytes responded differently with respect to induction of apoptosis. Therefore, at least under challenge, there is no complete compensation between the two NRPs. Both serum starvation (data not shown) or UVB irradiation resulted in significantly higher rates of apoptosis when NRP1 was absent. Under physiological conditions UVB is the more relevant inductor of apoptosis. Therefore, we continued our experiments with UVB irradiation.

Both UVA (320–400 nm) and UVB (280–320 nm) light are able to induce DNA damage in keratinocytes, but the mechanism of action differs. Whereas the higher energetic UVB irradiation causes mainly direct mutations (especially cyclobutane pyrimidine dimers, CPDs), UVA acts indirectly by increasing intracellular levels of reactive oxygene species (ROS), which in turn lead to 8-hydroxyguanine and finally to G-T transversion [33], [34]. Primarily UVB and to a lesser extent UVA can activate the keratinocyte apoptotic machinery. This is accomplished simultaneously by the extrinsic and the intrinsic way. On the cellular level, the activation of p53 and the reduction of pro-survival signals from activated MAP-kinase pathways are the hallmarks of UV-induced apoptosis, leading to inhibition of anti-apoptotic Bcl-2 proteins and activation of pro-apoptotic Bcl-2 members [29].

Our experiments with NRP1-deficient keratinocytes demonstrate that the mechanisms of UVB-induced apoptosis described above are influenced by the cellular expression of NRP1. In UVB-irradiated NRP1-deficient kertinocytes we measured reduced activation of Rac1, decreased Bcl-2 levels and higher amounts of phospho-p53. All these changes drive the cells towards apoptosis.

The observation that mice with keratinocyte-specific deletion of NRP1 did not show any spontaneous phenotype is in line with the fact that skin differentiation abnormalities are absent in any Bcl-2 family member knock-out reported so far [35]. From these results it can be concluded that in many cases alterations in the apoptotic system manifest only when cells or animals are exposed to stress like UVB irradiation or cytotoxic agents.

Since neuropilins are receptors for various different ligands like VEGF or class 3 semaphorins the question remains which ligands excert their effects on keratinocytes under physiological or pathological conditions, leading to decreased apoptosis in stress reactions. One possible candidate is VEGF. The fact that significant amounts of VEGF are secreted by epidermal keratinocytes is established knowledge since many years [36]. Keratinocyte-derived VEGF was believed to act primarily on endothelial cells of the underlying dermis, thereby helping to maintain the vascular plexus [37]. The expression and functional relevance of VEGF-receptors on keratinocytes has been discussed controversially for many years. Whereas early reports stated that VEGF-receptor tyrosine kinases are not expressed in the epidermis, more recent publications demonstrated that this assessments needs to be corrected. Wilgus et al. described the expression of VEGF-R1 on normal epidermal keratinocytes and during wound healing [38], whereas another publication demonstrated the expression of all three VEGF-Rs in human epidermis by immunofluorescence staining and in primary keratinocytes in vitro by RT-PCR and Western blotting [14].

The same publication by Man et al. also reported that VEGF was able to induce keratinocyte proliferation and migration. This effect could be inhibited by function-blocking antibodies to VEGF-R2, suggesting an autocrine stimulation loop of VEGF on keratinocytes. The influence of neuropilins on this loop was not investigated, but since the ability of NRPs to enhance the activation of VEGF-R2 by VEGFA is well documented [39], such an influence appears to be likely. Furthermore, a recent report defined an autocrine VEGF-NRP1 loop on cutaneous squamous cell carcinomas [40]. The authors provided evidence that this interaction is important for cancer cells to retain their stemness. Interestingly, regression of squamous cell carcinomas (SCCs) in mice could be achieved by blocking VEGF-R2. A role for VEGF as a growth factor for cutaneous SCCs had already been described before by Lichtenberger et al. [41]. Here it was demonstrated that the absence of VEGF impairs tumour formation not only by reduced tumour angiogenesis, but also by the absence of direct stimulatory effects on cancer cell proliferation. Taken together, the presence and functional relevance of VEGF receptor tyrosine kinases on normal and malignant keratinocytes has been demonstrated and it can be speculated that NRP1 has a role to enhance VEGF effects on these cells.

This assumption is supported by several publications demonstrating the influence of VEGF on apoptosis. Concerning non-malignant cells, anti-apoptotic effects of NRP1 in response to binding of VEGF have been reported for cell types like mesenchymal stem cells, neurons or cancer cells [13], [19], [20]. Our data demonstrate that NRP1 is implicated in the suppression of UV-induced apoptosis of normal epidermal keratinocytes. The physiological function would be to prevent excessive apoptosis in response to environmental hazards like high dose UV-irradiation. Further studies are needed to identify the NRP1-ligands and precise intracellular signal transduction cascades responsible for this function.
